# Time series analysis of dengue incidence in Guadeloupe, French West Indies: Forecasting models using climate variables as predictors

**DOI:** 10.1186/1471-2334-11-166

**Published:** 2011-06-09

**Authors:** Myriam Gharbi, Philippe Quenel, Joël Gustave, Sylvie Cassadou, Guy La Ruche, Laurent Girdary, Laurence Marrama

**Affiliations:** 1 Ecole Pasteur-Cnam de Santé Publique, Paris, France; 2 Regional office of the French Institute for Public Health Surveillance (Cire Antilles - Guyane), Fort-de-France, Martinique, France; 3 Department of Health and Social Development, Vector Control Department, Guadeloupe, France; 4 International and Tropical Department, French Institute for Public Health Surveillance (InVS), Saint-Maurice, France; 5 University of the French West Indies and of French Guyana, Guadeloupe, France; 6 Research Unit "Epidemiology and Transmission of Emerging diseases", Institut Pasteur of Guadeloupe, Guadeloupe, France

**Keywords:** Dengue fever, Time series analysis, SARIMA models, Forecasting, Climate, Guadeloupe, America region

## Abstract

**Background:**

During the last decades, dengue viruses have spread throughout the Americas region, with an increase in the number of severe forms of dengue. The surveillance system in Guadeloupe (French West Indies) is currently operational for the detection of early outbreaks of dengue. The goal of the study was to improve this surveillance system by assessing a modelling tool to predict the occurrence of dengue epidemics few months ahead and thus to help an efficient dengue control.

**Methods:**

The Box-Jenkins approach allowed us to fit a Seasonal Autoregressive Integrated Moving Average (SARIMA) model of dengue incidence from 2000 to 2006 using clinical suspected cases. Then, this model was used for calculating dengue incidence for the year 2007 compared with observed data, using three different approaches: 1 year-ahead, 3 months-ahead and 1 month-ahead. Finally, we assessed the impact of meteorological variables (rainfall, temperature and relative humidity) on the prediction of dengue incidence and outbreaks, incorporating them in the model fitting the best.

**Results:**

The 3 months-ahead approach was the most appropriate for an effective and operational public health response, and the most accurate (Root Mean Square Error, RMSE = 0.85). Relative humidity at lag-7 weeks, minimum temperature at lag-5 weeks and average temperature at lag-11 weeks were variables the most positively correlated to dengue incidence in Guadeloupe, meanwhile rainfall was not. The predictive power of SARIMA models was enhanced by the inclusion of climatic variables as external regressors to forecast the year 2007. Temperature significantly affected the model for better dengue incidence forecasting (p-value = 0.03 for minimum temperature lag-5, p-value = 0.02 for average temperature lag-11) but not humidity. Minimum temperature at lag-5 weeks was the best climatic variable for predicting dengue outbreaks (RMSE = 0.72).

**Conclusion:**

Temperature improves dengue outbreaks forecasts better than humidity and rainfall. SARIMA models using climatic data as independent variables could be easily incorporated into an early (3 months-ahead) and reliably monitoring system of dengue outbreaks. This approach which is practicable for a surveillance system has public health implications in helping the prediction of dengue epidemic and therefore the timely appropriate and efficient implementation of prevention activities.

## 1. Background

Dengue is a human arbovirus disease transmitted by the female mosquito of the genus *Aedes*, mainly *Aedes aegypti and Ae. albopictus *[1]. Dengue, the most frequent arthropod-borne viral disease, is prevalent in tropical and subtropical regions. Two major clinical forms of dengue illness involve the mild form of dengue fever and severe form mostly characterized by plasma leakage with or without haemorrhage [2].

Two-fifths of the world population (about 2.5 billion people) is at risk of dengue infection. The prevalence of this disease has grown dramatically in the recent decades. Between 50 and 100 million people are infected each year worldwide and more than 500,000 are hospitalized [3]. The average annual incidence was multiplied by thirty in the last fifty years. Incidence of dengue haemorrhagic fever (DHF) is increasing in many tropical regions inducing 20,000 deaths per year, mostly among children under 15 years [4].

In the Caribbean and Latin America countries, the reintroduction and dissemination of *Ae. aegypti *were observed in the 1970s, after the reduction of vector control interventions initiated in the 1960s. Since that time, regular outbreaks occured with a cycle of 3-5 years, combined with an increase in severe forms, particularly DHF [5]. In some areas, as the French Overseas Territories of the Americas (FOTAs: French West Indies, Martinique and Guadeloupe, and French Guyana), the epidemiology of dengue is moving from an endemo-epidemic situation towards a hyper-endemic situation [6]. Over the decade 1997-2007, the FOTAs had four major epidemics (1997, 2001, 2005 and 2007) each linked to the circulation of one or two predominant serotypes. These outbreaks usually last 4-6 months and may affect up to 5% of the population. The epidemiological dynamics observed over this decade in the FOTAs raises fears of a move towards a situation comparable to that currently in South East Asia and dengue could become one of the leading causes of hospitalization, especially for children. During the last two epidemics of dengue in Guadeloupe (400,500 inhabitants in 2007), the number of clinical cases that led to a medical consultation were respectively 11,500 in 2005 (0.4% of severe cases; serotype 4 was predominant) and 19,000 in 2007 (0.8% of severe cases; serotype 2 predominant).

Dengue is endemic in all surrounding countries with the four serotypes circulating in the region within a period of ten years. Countries or territories with the highest number of reported dengue cases were Puerto Rico, the Dominican Republic, Martinique, Trinidad and Tobago and French Guiana.

Population movement is an important factor in the virus dissemination. It contributes to carry new virus strains but it also participates to introduce non immune subjects in an endemic area. Dengue outbreaks may occur when a high proportion of naïve subjects are concentrated in the same area. As the social and economic impacts are worsening [7,8] and outbreaks are increasing, it becomes urgent to reinforce an integrated management for the surveillance, control and prevention of dengue [9]. One key aspect of this strategy is the ability to predict the occurrence of dengue outbreaks.

An early warning of dengue outbreaks could improve the efficiency of vector control campaigns and help to target prevention actions. Such early interventions could delay or spread out the epidemic, thus reducing its impact on health system. Health facilities could adapt their response in terms of availability of beds and mobilization of human and material resources [10]. Dengue morbidity and mortality would be minimized through earlier and more appropriate public health response.

Many complex mathematical models have been developed to predict the occurrence, dynamics and magnitude of outbreaks using a combined environmental and biological approach. Various parameters have been used [11,12] such as climatic data [13,14], vector characteristics [15,16], availability of breeding sites [14], viral serotypes circulating, immune status of host populations [13] or demographics data [17]. Meteorological conditions are considered as some of the most important factors of dengue in outbreaks occurrence [18]. Many studies have highlighted the relationship between climate and dengue transmission. The increase of temperature has been found associated with dengue in Thailand [19,20], Indonesia [21], [22] and [23], Singapore [24], Mexico [25] and Puerto Rico [26]. Elevated humidity with high mosquito density increased the transmission rate of dengue fever infection in southern Taiwan [27]. Meanwhile, a large amount of rainfall has been linked to dengue fever in Indonesia [23], Trinidad [28], Venezuela [29], Barbados [30] and Thailand [20].

These models, requiring important human and logistical resources for data collection and implementation, are difficult to be used for a continuous surveillance system and for early detection, particularly in developing countries. Therefore, it is important to develop a surveillance system based on a model that includes data as explanatory variables [31], respecting the following steps:

- To develop a model that correctly identifies and quantifies the relationship between dengue and climatic variables;

- To reduce the model to its most parsimonious form so that human and material resources could be focused on the collection of essential data;

- To ensure that the surveillance system is ongoing and sustainable and able to record cases at regular intervals (weekly or monthly).

The surveillance system in Guadeloupe is currently operational for the detection of early outbreaks of dengue. The aim of the study is to improve this surveillance system by assessing a modelling tool including meteorological data to predict the occurrence of dengue epidemics few weeks or months ahead (duration consistent with greater anticipation of outbreaks). This predicable model would be used to help an efficient dengue control.

We propose to develop Seasonal Autoregressive Integrated Moving Average (SARIMA) models using time series analysis of dengue incidence. Such models are particularly interesting when there are time dependences between each observation [32]. The assumption that each observation is correlated to previous ones makes it possible to model a temporal structure, with more reliable predictions, especially for seasonal infections [33-36], than those obtained by other statistical methods. SARIMA models have been successfully used in epidemiology to predict the evolution of infectious diseases, such as malaria and hepatitis A [35], deaths due to influenza [37] and pneumonia [33]. Moreover, these models allow the integration of external factors, such as climatic variables, that may increase their predictive power [38,39].

A prediction approach has been developed in this study using data from Guadeloupe. It could be implemented regionally or internationally, if it is evaluated and validated in other part of Caribbean and Latin America (beginning with others FOTAs).

## 2. Methods

### 1. Settings

Guadeloupe, with a surface of 1,434 km^2^, is an island located in the Caribbean, between the Tropic of Cancer and the Equator (latitude 15°57'-16°31' North and longitude 61°10'-61°48' West). The climate is tropical with two distinct seasons: a dry season from January to June, characterized by relatively low rainfall and a wet season from July to December.

### 2. Data Collection

This study covers the period from 2000 to 2007 for dengue incidence and meteorological data. During the study period, stable dengue control programs were implemented each year in Guadeloupe.

The sentinel network was set up from 1983 by local health authorities in association with Institut Pasteur in Guadeloupe. Until 2004, dengue-like syndrome reporting was linked to laboratory confirmation done by Institut Pasteur [40]. Then the monitoring of dengue-like syndromes was going on with sentinel physicians but was separated from confirmed case surveillance, as more and more laboratories were involved in diagnostic confirmation. Nowadays, epidemiologic surveillance system of dengue in Guadeloupe is based on 3 main indicators collected independently: (1) weekly number of dengue-like syndromes (suspected dengue cases) collected from sentinel general practitioners (GPs) for early detection and measurement of magnitude of epidemics; (2) weekly number of confirmed cases of dengue from all laboratories (hospital or not) for confirmation of virus circulation; (3) monthly number of confirmed hospitalized cases from hospital wards for measurement of epidemics severity. Thus, laboratory-confirmed cases, which is a less efficient indicator to monitor the dynamics of an epidemic, and syndromes dengue-like counted for surveillance are not directly linked. In this study, the dengue incidence was calculated from the weekly number of dengue-like syndromes and data from the Institut Pasteur were only used for validation purpose of this incidence.

The number of sentinel GPs, as well as the representativeness of the network in terms of GP's activity, has progressively improved to reach a quite good representativeness (it represents 12% of all GPs in Guadeloupe in 2008) with a high weekly participation rate (85% on average in 2008). Nevertheless, it could be qualitatively improved for a better adequacy with the geographical distribution of the population [41]. Dengue cases reported by practitioners are mainly non-severe; severe cases treated in hospitals are not included but represent less than 1% of dengue cases.

A suspected dengue case is defined as a patient with less than seven days of fever (≥38.5°C) without evidence of other cause of infection and with at least one symptom of pain (headache, retro-orbital pain, myalgia, arthralgia, back pain).

Each week, the total number of dengue cases collected is extrapolated to the whole island using for adjustment the ratio "medical activity of sentinel GPs present during the week"/"medical activity of all the GPs in Guadeloupe". Weekly incidence rates were calculated using demographic data for Guadeloupe from the National Institute of Statistics and Economic studies. Missing data were replaced using a simple exponential smoothing method. The time series were adjusted by adding the constant of one to the data. This procedure did not change the temporal structure of the series and allowed the use of a logarithmic transformation.

The meteorological station of Raizet (the international airport of Guadeloupe) records meteorological variables every day: cumulative rainfall (mm), relative humidity (%), minimum, maximum and average temperature (°C). The availability of these data explains the use of these climatic variables in our study. These data, aggregated on a weekly basis, cover the study period without any missing values.

### 3. Processing and data analysis

A SARIMA model was adjusted to the data of dengue incidence from 2000 to 2006, using the Box and Jenkins approach developed in 1974 [32,34,35,42,43]. This model allowed predicting dengue incidence for the year 2007.

Box-Jenkins method is a four-step process:

Firstly, the variance was stabilized using an appropriate transformation (logarithmic, square root or inverse transformation), based on a mean-range plot analysis as a relevant decision tool. The mean was also stabilized by differencing according to the profile of the original time series. A seasonal component was removed by a seasonal differencing: Zt - Zt-s (Zt = values of the time series at time t and Zt-s = values of the time series at time t-s with s corresponding to the seasonality) and the trend was removed by a regular differencing: Zt - Zt-1 (Zt-1 = values of the time series at time t-1 week). The seasonality as well as the trend are highlighted by plotting the original time series. The presence of these two components determine the choice of a SARIMA model equation:  where Φ_*P*_(*B*^*S*^) is the seasonal autoregressive (AR) operator, Φ_*P*_(*B*) AR the operator, Θ_*q*_(*B*) the moving average (MA) operator, Θ_*Q*_(*B*^*S*^) the seasonal MA operator, (1 - *B*)^*d *^and (1 - *B*^*S*^)^D ^the ordinary and seasonal difference components, *a*_*t *_the white noise, and *y*_*t *_the dependant variable.

The choice of the order of integration (D, d) parameters is based on the plot of integrated time series and based on comparison of their standard deviation; the series having the more stabilized mean is selected.

Secondly, the temporal structure of the series, i.e. order of seasonal and non seasonal AR (P, p), MA (Q, q) parameters, were determined. Several tools are available:

- To identify the order of MA and AR parameters, the structure of temporal dependence of stationary time series is assessed respectively, by the analysis of autocorrelation (ACF) and partial autocorrelation (PACF) functions.

- To select the model, with fewer parameters that fits the data best, the Akaïke Information Criterion (AIC) is used.

- To validate the final model, its residuals are analyzed by the Ljung-Box test. Residuals must be equivalent to white noise.

Thirdly, model parameters were estimated by the maximum likelihood method.

Finally, predicted data for 2007 were compared with observed data in order to validate the model. The average error was calculated using the RMSE (Root Mean Square Error) equals to: with *Y*_*t *_the observed value and  the predicted value at t time and N the number of observations.

The predictions and their 95% confidence intervals were estimated for 2007 using the best model fitting the data for 2000-2006. Three different methods were compared; the first consisted to predict 2007 data with a one year (52 weeks) lag; the second and third methods were iterative approaches. They consisted to predict dengue incidence, with three (13 weeks) and one month (4 weeks) lags, respectively. After the prediction of the first period, the observed data for this period were included in the database in order to update the model and estimate the predictions for the second period. The same were used to complete the predictions for the whole year 2007.

Once the univariate model was selected, the multivariate models including external regressors could be elaborated. Meteorological variables were used to improve the predictive power of the model. In order to include these explanatory variables, cross-correlation graphics (Pearson test) between data of dengue incidence and climatic variables for a 16 weeks period were performed.

As the use of cross-correlations on original time series is not recommended, they were performed on the residuals of the models obtained by applying a SARIMA model to each series [44]. Climatic variables significantly associated to dengue incidence were tested as predictors in a multivariate SARIMA model. The model equation is , where X is the external variable.

This model was able to give predictions with a lag of one, three or twelve months, depending on the approach selected during the univariate process. The predictive power of the models was estimated with the RMSE (a significant decrease of RMSE denotes an improvement of the model) and the Wilcoxon signed-ranks test which was used for significance assessment.

The statistical software R (version 2.9.0) and Statgraphics were used for all analyses. Statgraphics allowed a first approach of the problem. R had confirmed all items found on Statgraphics. The graphics were performed with R.

## 3. Results

**Figure 1:**
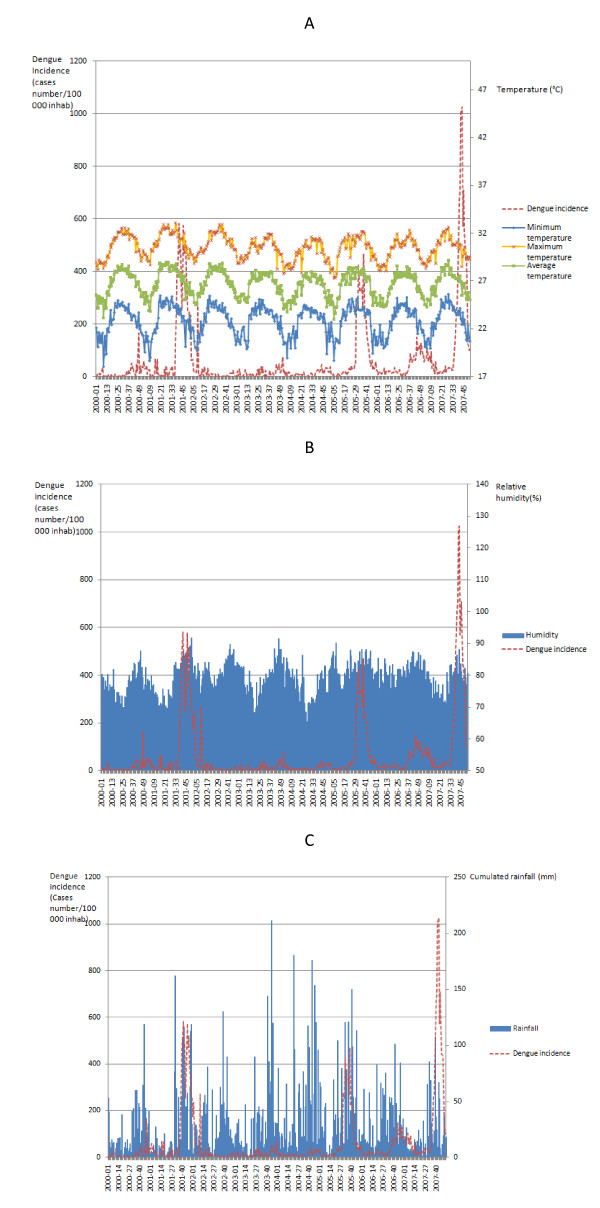
**Dashed red line: Weekly incidence rates of dengue (per 100,000) in Guadeloupe from January 2000 to December 2007 compared to crude meteorological variables for the same period**: A) minimum temperature (blue diamond), maximum temperature (yellow cross) and average temperature (green square); B) relative humidity (blue area); C) weekly cumulated rainfall (blue solid line).

The plot of the observed dengue incidence (Figure 1) showed three major outbreaks in Guadeloupe (late 2001 - early 2002, mid 2005 - early 2006, mid 2007 - late 2007). The bivariate analysis between crude climatic variables and dengue incidence shows that the three major outbreaks were correlated to a slight decrease of temperature (Figure 1A) and to an increase in relative humidity (Figure 1B). Dengue incidence was not clearly correlated to weekly cumulated rainfall (Figure 1C). An annual seasonality is identified for all these meteorological variables.

**Figure 2:**
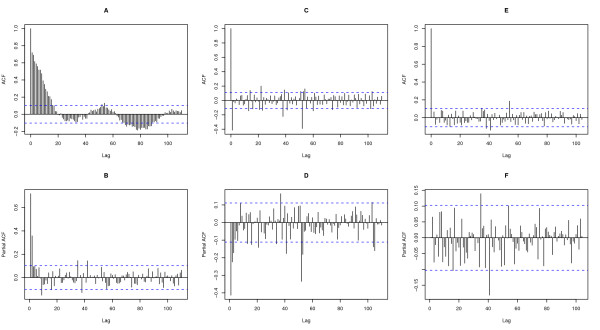
**A and B) Autocorrelation function (ACF) and Partial ACF (PACF) plot of original dengue incidence**. C and D) ACF and PACF plot of integrated dengue incidence. E and F) ACF and PACF of residuals after applying a SARIMA (0, 1, 1) (0, 1, 1)_52 _model. The X-axis gives the number of lags in weeks and, the y-axis, the value of the correlation coefficient comprised between -1 and 1. Dotted lines indicate 95% confidence interval.

**Figure 3:**
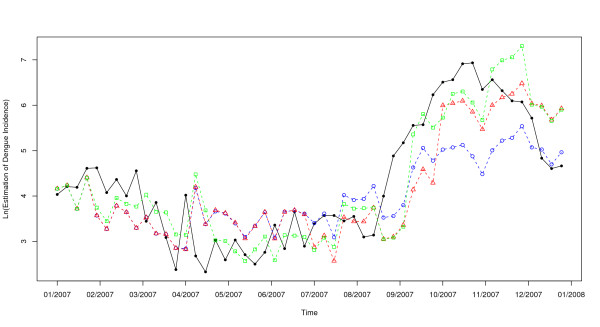
**Natural logarithm of dengue incidence in Guadeloupe for 2007**. Solid line (filled circle): observed values during the period. Dashed lines: univariate SARIMA (0,1,1) × (0,1,1)52 model; blue (circle): 52 weeks ahead values; red (triangle): 13 weeks ahead values; green (square): 4 weeks ahead values.

In the first step of the dengue time series analysis, it has been necessary to stabilize the variance of the series by natural logarithm transformation. This transformation had the lowest dispersion. To stabilize the mean, seasonal and regular differencing were applied. The plots of ACF and PACF (Figures 2A and 2B) showed the temporal dependence of the dengue incidence and confirmed the need to use a SARIMA model with seasonal (P, D, Q) and non-seasonal (p, d, q) parameters. After differencing, a significant cut-off at one week-lag and another at lag-52 weeks were observed on the plot ACF (Figure 2C). These two cut-offs were less marked on the plot PACF (Figure 2D) and evolve more gradually over the time, compared to the plot ACF. Therefore, the following univariate multiplicative SARIMA (0,1,1)(0,1,1)_52 _model was the best to fit the dengue incidence (AIC = 951, Table 1). The analyses of residuals on ACF and PACF plots (Figure 2E and 2F) assessed the absence of persistent temporal correlation. The Ljung-Box test confirmed that the residuals of time series were statistically not dependent (p-value > 0.05). The selected SARIMA model fitted observed data from 2000 to 2006 (RMSE = 0.914). For 2007, the 3 methods were compared (Figure 3). The 4 weeks-step approach showed the smallest difference between observed and predicted values (RMSE = 0.76) when compared to the 52 weeks-step approach (RMSE = 0.98) and to the 13 weeks-step approach (RMSE = 0.85). However, the difference between residuals predicted 13 weeks-ahead and those predicted 4 weeks-ahead was not statistically significant (Wilcoxon signed-ranks, p-value = 0.48). The predictions for the 3 following months were the best compromise for helping the health authorities to take measures to mitigate transmission, morbidity and mortality.

**Table 1:** Coefficients, standard errors, t statistic and P-value of the parameters of the SARIMA(0,1,1)(0,1,1)_52 _model estimated by maximun likelihood

Parameters	Coefficients	Standard error	T statistic	*P*-value
Non seasonal MA (q)	-0.6092	0.0481	-12.66	9.56e-37*

Seasonal MA (Q)	-0.8858	0.1875	-4.72	2.32e-06*

MA: moving average, *:p-value < 0.05 significant.

To include climatic variables (time series) as external variables in the univariate model, a SARIMA model was applied to all time series (Table 2). Cumulative rainfall and relative humidity variables were log transformed. A regular differencing was applied for all variables except for minimum temperature. A seasonal differencing was also applied for all variables except for relative humidity. The residuals were kept for the multivariate analyses.

**Table 2:** Characteristics of SARIMA models for climatic variables: coefficients, standard errors of residuals, AIC: Akaike information criterion, p-value after Ljung-Box test of residuals

Climatic variables	**SARIMA (p,d,q) (P,D,Q)**_*S *_**model**	AR1	MA1	SMA	Sd (residuals)	AIC	p-value (Ljung-box test)
**Accumulated rainfall**	(1,1,1) (0,1,1)_*52*_	0.154*	-0.980*	-1.00*	0.85	942.78	0.32 (NS)

**Relative humidity**	(1,1,1) (0,0,0)_*52*_	0.207*	-0.632*	_	0.039	-1320	0.307 (NS)

**Minimum temperature**	(0,0,1) (0,1,1_)*52*_	0.249*	_	-1.00*	0.67	785.69	0.61 (NS)

**Maximun temperature**	(1,1,1) (0,1,1)_*52*_	0.439*	-0.918*	-1.00*	0.473	576.63	0.566 (NS)

**Average temperature**	(1,1,1) (0,1,1)_*52*_	0.310*	-0.927*	-0.897*	0.473	556.92	0.566 (NS)

AR: autoregressive, MA: moving average, SMA: seasonal moving average, *:p-value < 0.05 significant.

**Figure 4:**
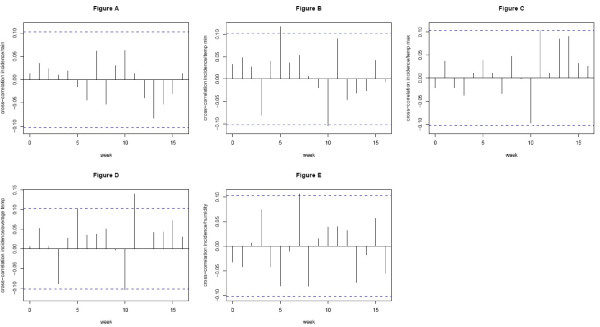
**Cross correlation functions between dengue fever cases and meteorological variables after applying SARIMA models**. The x-axis gives the number of lags in weeks. Dotted lines indicate 95% confidence interval. Only positive lags are taken into account a) Accumulated rainfall b) Minimum temperature c) Maximum temperature d) Average temperature e) Relative humidity.

Then, correlations between residuals of dengue incidence and those of meteorological variables over a range of 16 weeks-lags were analyzed. Climatic variables identified as the most correlated to dengue incidence were included one by one, because of their strong interrelationship, to test their influence on the model. The following explanatory variables were the most positively correlated with dengue incidence: lag-5 minimum temperature (Pearson correlation: r = 0.118, p-value = 0.025), lag-11 average temperature (r = 0.141, p-value = 0.007) and lag-7 relative humidity (r = 0.107, p-value = 0.04), (Figure 4). Meanwhile, rainfall was not correlated with dengue incidence over a range of 16 weeks-lags (r < 0.06, p-value = non significant).

**Figure 5:**
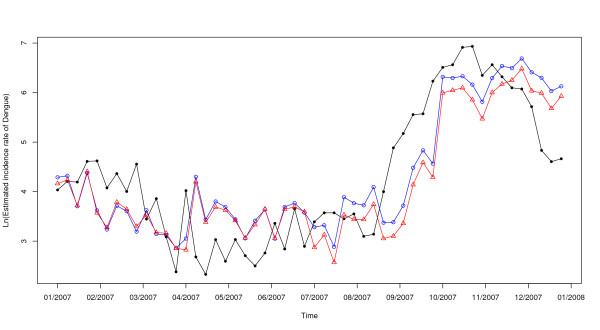
**Natural logarithm of dengue incidence in Guadeloupe for 2007**. Solid line (filled circle): observed values during the period. Red (triangle): univariate SARIMA (0,1,1) × (0,1,1)52 model's 13 weeks ahead values. Blue (circles): multivariate SARIMA (0,1,1) × (0,1,1)52 model's 13 weeks ahead values with minimum temperature lag-5.

**Figure 6:**
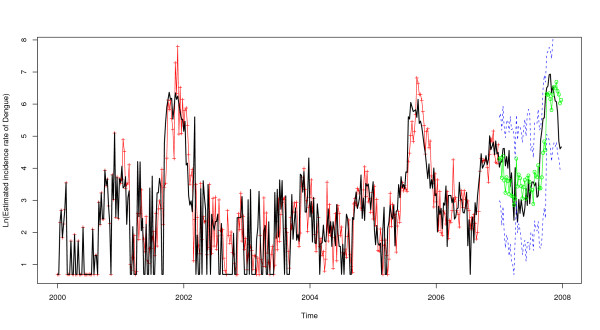
**Natural logarithm of dengue incidence in Guadeloupe for the 2000-2007 period**. Solid line: observed values during the period. Red (crosses): Fitted values from 2000 to 2006 and green (circles): predicted values for 2007 with multivariate SARIMA (0,1,1) × (0,1,1)52 model's 13 weeks ahead values with minimum temperature lag-5 as an external regressor and their 95% prediction intervals (Blue dashed lines).

SARIMA models (0,1,1)(0,1,1)_52 _including external independent variables, either the lag-5 minimum temperature or the lag-11 average temperature, were statistically relevant. These two variables influenced individually the model (Table 3). The predictions for 2007 per period of 3 months were improved after the introduction of either the minimal temperature at lag-5 weeks or the average temperature at lag-11 weeks (RMSE = 0.72 and 0.74, respectively, versus 0.85 for the univariate model) (Figures 5 and 6). However, these differences were not statistically significant (Wilcoxon signed-rank test: p-value = 0.364 and 0.407, with lag-5 minimum temperature and lag-11 average temperature, respectively).

**Table 3:** Characteristics of univariate and multivariate models using climatic variables the most correlated to dengue incidence: Coefficients, standard error and p-value of parameters, AIC, RMSE for predictions of the year 2007 3 months-ahead, * p-value < 0.05 significant

SARIMA model	Coefficients	Standard error	T- statistic	P-value	AIC	RMSE
**Univariate model**					951	0.85

**Relative humidity _lag7**	1.445	1.423	1.016	0.309	951	0.76

**Minimum temperature _lag5**	0.108*	0.068	1.593	0.030	950	0.72

**Average temperature _lag11**	0.228*	0.102	2.235	0.025	948	0.74

## 4. Discussion

We develop a model to improve the surveillance system and thus to help efficient dengue control. In the study, the predictions for the year 2007 per period of 3 months were improved after the introduction of the minimal temperature at lag-5 weeks or the average temperature at lag-11 weeks, but without reaching statistical significance when we compare univariate and multivariate SARIMA models. However, multivariate model can provide better prediction and might be implemented in routine for dengue surveillance in Guadeloupe.

This model, if tested in practice and validated with other set of data, might be useful for the evaluation of new intervention strategies introduced into this island.

The time series analysis has allowed the development of robust and reliable SARIMA models with a correct level of validity that fits dengue incidence data collected in Guadeloupe from 2000 to 2006. It showed that the predictions over a period of 3 months for the year 2007 were the best way to implement appropriate prevention programs.

Moreover, the study demonstrated a positive correlation between dengue incidence and climatic variables such as relative humidity, minimum temperature and average temperature.

The predominant effect of these variables was observed after a 7 weeks-lag for the relative humidity, 5 weeks-lags for the minimum temperature, and 11 weeks-lags for the average temperature. Observed correlations are weak (below 0.2) since they are measured on residuals after an adjustment on the trend and the seasonality. However, they are statistically significant, which means the test is powerful, probably due to the high numbers of observations in the study.

These models present some limits. Firstly, the time series analysis requires homogeneous data over long periods, which are often difficult to obtain (i.e. the surveillance system in Guadeloupe was improved after 2004 providing heterogeneous data during the period of change). Ongoing dengue surveillance in Guadeloupe will allow local health authorities to improve this model since the reliability of predictions increases as time series become longer.

Secondly, the crude relationship between dengue incidence and climatic variables (Figure 1) shows that modification of the weather does not necessarily affect dengue epidemics while the use of SARIMA modelling, which is more relevant, points out a significant effect of these variables. Some other climatic events not available for the study have not been analyzed: (1) vapor pressure which is a measure of relative humidity by satellite remote sensing [45] is less pertinent than humidity in our geographical situation because of the poor resolution in a small territory surrounded by water as the Guadeloupe Island; (2) the link between ENSO (El Niño-Southern Oscillation) and dengue incidence through mechanisms modifying the vector, the virus and the human behavior [46,47] should be studied in the future to support our results. The delay between changes associated with ENSO and the increase in the dengue incidence could be as long as several months [46]; (3) the effect of hurricane in dengue virus circulation is not clear and difficult to take into account in the analyses. However, one of our teams (the Cire Antilles Guyane) tried to assess by a score the impact of the Hurricane "Dean" on the risk of dengue [48]. A deeper study should be done to evaluate correctly the effect of these strong events on the dengue incidence. Other non climatic factors might explain outbreaks of dengue such as (i) geographical characteristics of the study area (i.e. housing density, climate and agriculture works) and (ii) virus characteristics (serotype or virulence), but could not be explored in our study because of the non temporal format of these variables. However, the serotype should not affect so much the occurrence of epidemics because of the endemicity in the Caribbean region and the circulation of the four serotypes within a period of ten years. Most of the population has already been exposed to at least one serotype of dengue virus.

Finally, this model, which is global for Guadeloupe as a whole and not local by district, does not take into account geographical disparities in this island.

Our results are consistent with those of other studies dealing with the effect of climate on dengue outbreaks. These studies have highlighted that many climatic variables play a key role in dengue transmission. Although rainfall plays an ambiguous role on dengue incidence, temperature and relative humidity affect this transmission in several ways.

The potential impact of rainfall could either increase the transmission of vector-borne diseases, by promoting the proliferation of breeding sites, or eliminate breeding sites by flooding them [18,49,50].

More than rainfall, temperature affects the dynamics of *Ae. aegypti *by reducing the duration of the gonotrophic cycle [51], estimated at 6 to 7 days [52-54]. A proportional inverse effect is also observed in *in vitro *studies with duration range from 39.7 to 7.2 days for temperatures varying from 15°C to 35°C [55]. Moreover, temperature can increase the number of blood meals pro vector during each cycle amplifying significantly the level of transmission [56].

Relative humidity is another key factor that influences mosquitoes at different stages, especially during mating and egg laying. The combined effect of temperature and humidity influences significantly the number of blood meals and can also increase the survival rate of the vector, so that the probability it becomes infested during its life increases [57-59]. Moreover, higher temperatures make viral spread easier by reducing the extrinsic incubation period of *Ae. aegypti *(*in vitro*: 12 and 7 days, at 30°C and between 32°C and 35°C, respectively [60]). In the literature, relative humidity and temperature are two important variables, more than rainfall. However, in Guadeloupe between 2000 and 2007, the relative humidity was a very stable parameter (mean = 79.3%; IC95% [78.9, 79.7]). This very low range of variation might explain the low statistical power for this variable and the difficulty to point out a significant effect of the relative humidity in the SARIMA model.

Furthermore, the delayed effect of climatic variables on dengue incidence might be explained by climatic factors which do not influence directly the incidence of dengue but only indirectly through their effect on the life-cycle dynamics of both vector and virus. From mosquito hatching to human case appearance, several successive phases occur resulting in global cumulative lags observed in our study. These phases include larval and pupa development (10 to 21 days), gonotrophic cycle (3 to 7 days pro cycle), extrinsic incubation in mosquitoes (7 to 15 days), incubation in human (1 to 12 days). Depending on the respective lag between the biological cycle or mosquito life-stage and the clinical symptoms, the lag between weather data and incidence data will differ. The lag is expected to be shorter for minimum temperature that is usually associated with adult mosquito's mortality, longer for high relative humidity, both related to adult survival and hatching. On the other side, the mean temperature is involved in all biological cycles of *Ae. aegypti *that take more time to influence the dengue incidence.

Many studies have stressed a lag of several months. In Thailand, the dengue incidence was positively correlated with the average temperature at lag 3-4 months [19]. In Taiwan, there was a significant positive correlation with the maximum temperature at lag 1-4 months, the minimum temperature at lag 1-3 months and the relative humidity at lag 1-3 months [38]. In Brazil, positive associations were found between the minimum and maximum temperatures and dengue transmission at lag-0 [39]. And in the city of Guangzhou in China, the minimum temperature and relative humidity were positively correlated with the dengue incidence at lag-1 month [61].

Our results are consistent with all of these assumptions. We have shown that the increase in temperature and relative humidity were determining factors in predicting changes of the dengue incidence. On the contrary, rainfall did not appear to play a significant role. Actually, many breeding sites of *Ae. aegypti *are more dependent on human behavior than on rainfall for their constitution and persistence. That is the reason why local health authorities in Guadeloupe make people aware through sensitization campaigns since 2000. This might partly explain the lower impact of rainfall compared to other climatic variables on the dengue incidence.

We suggest that health authorities in other part of Caribbean or Latin America could introduce climatic variables in a local SARIMA approach to assess in another context the improvement of forecasting dengue incidence and outbreaks intensity.

The SARIMA modelling, used for predicting dengue incidence and outbreaks over a period of few months, must be considered as complementary from other modelling methods using non-epidemic data, as the Serfling regression model [62]. This model allows calculating a time-varying threshold, where an alert is generated if current data exceed the threshold. In Guadeloupe, such a modelling has been implemented since few years [63].

## 5. Conclusion

A reliable and robust model, that predicts the dengue incidence and outbreaks intensity over a period of 3 months, has been developed. This delay allows health authorities to launch an active vector control campaign, combining insecticide spraying and breeding sites destruction. Health facilities can also adapt patients care by increasing the number of available hospitalization beds if a dengue fever outbreak is predicted. This approach, which detects dengue epidemic risk and predicts outbreak intensity, fits very well with the current needs of the local health authorities. The model could contribute to dengue surveillance in Guadeloupe. The Cire Antilles-Guyane, department of public health in charge of surveillance data analysis, could update regularly the model, using data the most recently collected in the context of the ongoing and weekly dengue surveillance system. Therefore, they would obtain complementary information about outbreak risks.

## Competing interests

The authors declare that they have no competing interests.

## Authors' contributions

MG participated in the conception and the design of the study, performed the statistical analysis, participated in interpretation of data and in drafting the manuscript. PQ and SC participated in acquisition of data and revised the manuscript critically. JG has made substantial contributions to conception of the study. GLR participated in interpretation of data and revised the manuscript critically. LG helped to collect data. LM has proposed the research, made substantial contributions to conception and design of the study and coordination, participated in interpretation of data and has helped to draft the manuscript. All authors read and approved the final manuscript.

## Pre-publication history

The pre-publication history for this paper can be accessed here:

http://www.biomedcentral.com/1471-2334/11/166/prepub
